# Structural, Optical, and Magnetic Properties of Zn-Doped CoFe_2_O_4_ Nanoparticles

**DOI:** 10.1186/s11671-017-1899-x

**Published:** 2017-02-21

**Authors:** Tetiana Tatarchuk, Mohamed Bououdina, Wojciech Macyk, Olexander Shyichuk, Natalia Paliychuk, Ivan Yaremiy, Basma Al-Najar, Michał Pacia

**Affiliations:** 1 Department of Inorganic and Physical Chemistry, Vasyl Stefanyk Precarpathian National University, 57, Shevchenko Str., Ivano-Frankivsk, 76018 Ukraine; 2 Department of Physics, College of Science, University of Bahrain, PO Box 32038, Manama, Kingdom of Bahrain; 3 Faculty of Chemistry, Jagiellonian University, Ingardena Str., 3, 30-060 Kraków, Poland; 4 Faculty of Chemical Technology and Engineering, UTP University of Science and Technology, 3, Seminaryjna Str., 85-326 Bydgoszcz, Poland; 5 Department of Material Science and New Technology, Vasyl Stefanyk Precarpathian National University, 57, Shevchenko Str., Ivano-Frankivsk, 76018 Ukraine

**Keywords:** Spinel ferrite, Nanoparticles, Ferromagnetism, Vibrational modes, Energy band gap

## Abstract

The effect of Zn-doping in CoFe_2_O_4_ nanoparticles (NPs) through chemical co-precipitation route was investigated in term of structural, optical, and magnetic properties. Both XRD and FTIR analyses confirm the formation of cubic spinel phase, where the crystallite size changes with Zn content from 46 to 77 nm. The Scherrer method, Williamson-Hall (W-H) analysis, and size-strain plot method (SSPM) were used to study of crystallite sizes. The TEM results were in good agreement with the results of the SSP method. SEM observations reveal agglomeration of fine spherical-like particles. The optical band gap energy determined from diffuse reflectance spectroscopy (DRS) varies increases from 1.17 to 1.3 eV. Magnetization field loops reveal a ferromagnetic behavior with lower hysteresis loop for higher Zn content. The magnetic properties are remarkably influenced with Zn doping; saturation magnetization (M_s_) increases then decreases while both coercivity (H_C_) and remanent magnetization (M_r_) decrease continuously, which was associated with preferential site occupancy and the change in particle size.

## Background

Researchers have been interested in studying materials in their nanoscale dimensions due to their high surface area resulting to enhanced properties in comparison with the bulk materials counterpart [1–6]. Spinel ferrite (SF) materials with a general formula of AFe_2_O_4_, where A stands for metals as (Mn, Co, Ni, Mg, or Zn), are well known of their remarkable electrical, optical, and magnetic properties, especially in nanometer scale [7–9].

Doping with metal ions as (Zn, Co, Sr, and Gd) was aimed to improve the physicochemical properties of ferrite nanoparticles (NPs) essential for their applications such as photocatalysis [10, 11] in photodegradation of dyes and as antibacterial agents [12, 13], industrial applications [14], and electrochemical energy storage materials [15, 16]. Studies confirmed that doping influences the structural [17], optical [18], electrical [17, 19], infrared radiation properties [20, 21], and magnetic properties [22, 23].

For instance, crystallite size was shown to gradually increase from 12.6 nm for pure ZnFe_2_O_4_ to 21.17 nm for Mg-doped one (75%) [22]. The magnetization properties were found to be altered too, as the saturation magnetization (M_s_) and remanent magnetization (M_r_), at room temperature, increased from 19 to 8 emu/g for pure ZnFe_2_O_4_ prepared by combustion method to 45 and 16 emu/g, respectively, for 50% Mg-doped one. However, with further increase of Mg concentration, these values started to drop reaching 16 and 3.5 emu/gm, respectively, for 75% Mg-doped ferrite [22]. Magnetic properties measured at 77 K and were found to follow the same trend as observed at room temperature. The change in magnetic properties with the concentration of dopant was explained by the replacement of Fe ions and the dopant ions in the octahedral and tetrahedral sites according to their site preference. In general, magnetic properties are known to be strongly influenced by annealing [24]. Few reports predicted that oxide nanoparticles tend to undergo nucleation and growth of Fe ions as a result of electron beam annealing [25]. Such Fe nanoclusters are reported to cause large magnetoresistance due a combination of geometric and spin dependent scattering [26]. Moreover, Sr-doped ZnFe_2_O_4_ nanoparticles synthesized by microwave combustion were investigated in terms of structural and magnetic properties. Enhancement of coercivity (H_C_) with the concentration of Sr was linked to the lattice parameter that increased with the Sr-dopant concentration too. This effect was claimed to be caused by the expansion of the unit cell volume caused by doping with Sr ions (Sr has a higher ionic radius (*r* = 1.44 Å) compared with Zn (*r* = 0.83 Å)) [18]. Another study demonstrated very similar dependence: the increase of lattice parameter when SrFe_12_O_19_ NPs prepared by sol–gel method were doped with Ni and Zr ions [27]. Value of M_s_ was shown to be enhanced with the concentration of the dopant ions, while value of H_C_ was shown to decrease. This was related to the replacement of ions in spin-down states and to the grain size variations.

Among these ferrite NPs, CoFe_2_O_4_ is recognized for its significant chemical stability, high Curie temperature, and high magnetization [7, 8]. Studies on such materials involved analysis of the effect of Gd doping on the structural and magnetic properties of CoFe_2_O_4_ synthesized through wet chemical co-precipitation method [7]. X-ray diffraction (XRD) analysis confirmed the decrease of lattice parameter with the concentration of Gd^3+^, on the other hand, the crystallite size raised from 15 to 17 nm with the increase of doping ion concentration from 0 to 15%. A considerable reduction of both saturation magnetization (M_s_) and remanent magnetization (M_r_) from 91 to 29 emu/g, respectively, for pure CoFe_2_O_4_ to 54 and 15 emu/g, respectively, for CoFe_2_O_4_ doped with 15% Gd^3+^. This was referred to the large ionic radius of the dopant Gd^3+^ (*r* = 0.94 Å) compared to that of Co^2+^ (0.58 Å) leading to a preferential occupation of octahedral sites resulting in the disturbance of the ferromagnetic ordering thereby, leading to a lower magnetization [7]. A recent study showed a similar effect when CoFe_2_O_4_ NPs synthesized by sucrose-assisted combustion route were doped with Zn [23]; both M_s_ and M_r_ decreased with increasing Zn concentration which was also referred to the occupancy preference of octahedral and tetrahedral sites. Moreover, the coercivity (H_C_) was noticed to decrease too, from 126.5 to 26.3 kOe, due to the low anisotropy constant of the Zn^2+^ ions.

It is well known that the preparation technique has a direct influence on the nanoparticle’s shape and size and thus can affect the physical and chemical properties of nanostructures. The magnetic ferrite particles in the nanoscale regime can be synthesized by different methods like soft chemical methods such as co-precipitation, hydrothermal, sol–gel, etc. But the main advantage of co-precipitation method resides in providing particle size in the nanoscale regime with a high crystallinity. Such individual nanoparticles have a large constant magnetic moment and behave like a giant paramagnetic atom with a fast response to applied magnetic field with negligible remanence and coercivity (supermagnetic behavior). Nanoparticles can also result in a low saturation magnetization. These features make superparamagnetic nanoparticles very attractive for a broad range of applications in particular biomedical field. Therefore, the magnetic properties of nanoparticles highly depend upon the synthesis procedure.

Nanocrystalline CoFe_2_O_4_ with unique properties has potential applications in high frequency device, memory core, recording media, and in biomedical field. It is known that zinc ions (Zn^2+^) with diamagnetic nature are known for achieving good control over magnetic parameters in developing technologically important materials. Substitution of magnetic (Co^2+^) by a nonmagnetic (Zn^2+^) cations in spinel ferrite phase may induce important changes in their structural, optical, magnetic, and others properties, due to the distribution of cations in between the available A and B sites. However, a detailed study on the structural, elastic, optical, and magnetic properties of Zn^2+^-doped CoFe_2_O_4_ nanoparticles obtained by co-precipitation method in the widely region has not yet been reported so far. The aim of the present work is to synthesize nanoparticles of Co_1−x_Zn_x_Fe_2_O_4_ with *x* varying from 0 to 0.5 from metal salts by co-precipitation of hydroxides. The influence of Zn substitution on the structural, optical, and magnetic properties for this system has also been discussed.

## Methods

### Synthesis of Zn-Doped Cobalt Ferrites

The detailed description of synthesis route has been presented in our earlier work [28]. For the preparation of Co_1−x_Zn_x_Fe_2_O_4_ (*x* = 0, 0.1, 0.2, 0.3, 0.4, 0.5) samples through co-precipitation method, cobalt, zinc, and iron nitrates were taken in stoichiometric proportions and dissolved separately in distilled water. The as-prepared solutions were mixed and stirred intensely for 1 h to improve homogeneity. The solutions were subjected to constant heating at 80 °C under continuous stirring. Then, 4 M solution of NaOH was added slowly dropwise in required proportion. The black precipitate was formed and then was washed several times with distilled water, then heated at 100 ° C for 72 h for drying. The dried powders were heated at 800 ° C for 2 h and then were left to cooldown slowly to room temperature.

### Characterization Techniques

The crystal structure was checked by means of X-ray diffraction (XRD) method using diffractometer equipped with CuK_α_ radiation. The surface morphology was characterized by scanning electron microscopy (SEM) using JEOL JSM-T220A with an accelerating voltage of 20 kV. The particle size of powders was estimated by transmission electron microscope (TEM) operating at 75 kV. Samples were prepared by drop coating from alcohol dispersion on the copper grids using ultrasound. The elemental chemical composition was studied by energy dispersive spectroscopy (EDS) by means of REMMA-102-02 Scanning Electron Microscope-Analyzer (JCS SELMI, Ukraine). Fourier transmission infrared (FTIR) spectra were recorded in the wavenumber range 4000–350 cm^−1^ using Alpha-P FTIR spectrometer (Bruker) in ATR mode on diamond window with 256 scans at 6 cm^−1^ resolution. Each spectrum represents the average of six scans. UV–vis diffuse reflectance spectra were recorded using Shimadzu UV-3600 spectrophotometer equipped with an integrating sphere (diameter of 15 cm). BaSO_4_ was used as a reference. All samples were ground with BaSO_4_ (1:50) prior to measurements. Magnetic measurements (magnetization, remanence, coercivity) were performed using vibrating sample magnetometer (VSM) at room temperature under an applied field of ±10 kOe.

## Results and Discussion

### X-ray Diffraction Analysis

**Fig. 1:**
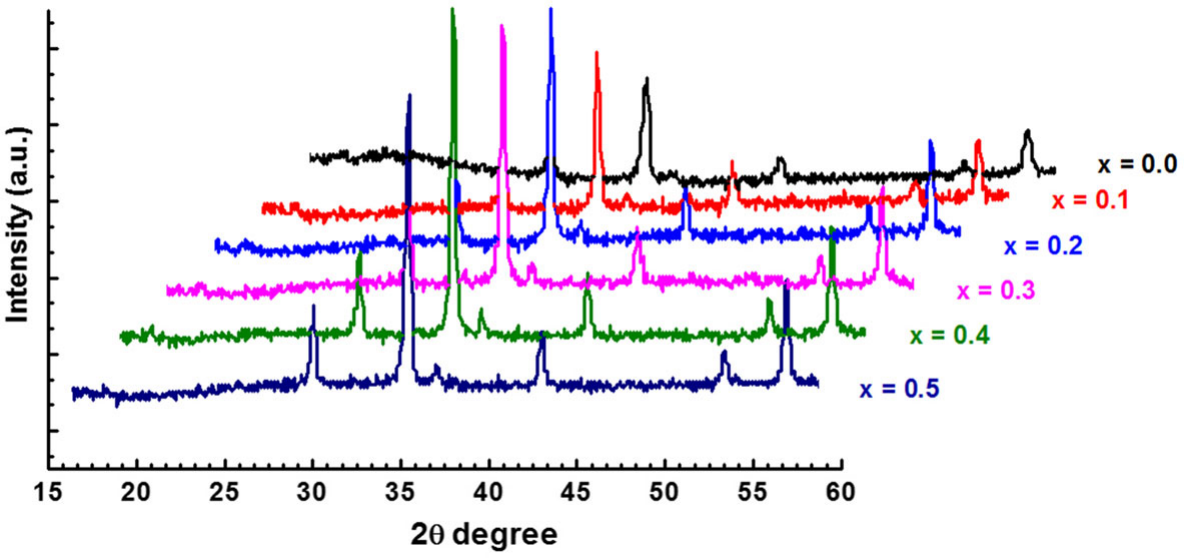
The X-ray diffraction patterns of the Co_1−x_Zn_x_Fe_2_O_4_ as function of Zn^2+^ content

X-ray diffraction (XRD) was performed on the powders calcined at 800 °C, and the XRD patterns of all the samples were shown in Fig. 1. The obtained patterns confirm the formation of a homogeneous single phase having cubic spinel structure with the space group Fd3m. The patterns show diffraction peaks of Co_1−x_Zn_x_Fe_2_O_4_ (*x* = 0.0, 0.1, 0.2, 0.3, 0.4, 0.5), corresponding to (111), (220), (311), (222), (400), (422), (511), and (440) reflections. All XRD patterns are analyzed by using the Rietveld method and FullProf program. The results show that the lattice parameter *a* slightly increases with Zn^2+^-doping content as shown in Table 1. The increase of *a* with *x* can be explained on the basis of the difference in ionic radii of Zn^2+^ and Co^2+^. The smaller ionic radius of Co (0.58 Å) was replaced by the larger ionic radius of Zn (0.6 Å) so the lattice parameter increased due to the expansion of the unit cell.

**Table 1:** Chemical composition, lattice parameter *a*_exp_, crystallite size *D*, and band gap of the Co_1−x_Zn_x_Fe_2_O_4_ spinels

*x* (Zn^2+^)	Chemical composition	*a*_exp_ (Å)	Crystallite size *D* (nm)				Band gap (eV)
			Scherrer method	W-H method	SSP method	TEM	
0.00	CoFe_2_O_4_	8.3536	27	26	25	51	1.17
0.10	Zn_0.1_Co_0.9_Fe_2_O_4_	8.3794	36	42	31	46	1.30
0.20	Zn_0.2_Co_0.8_Fe_2_O_4_	8.3897	51	49	38	53	1.28
0.30	Zn_0.3_Co_0.7_Fe_2_O_4_	8.3982	54	54	43	55	1.34
0.40	Zn_0.4_Co_0.6_Fe_2_O_4_	8.4016	55	56	47	69	1.32
0.50	Zn_0.5_Co_0.5_Fe_2_O_4_	8.4068	53	58	46	77	1.31

By dividing Kα-doublet of each observed peaks, it is found that the peaks would be implicitly described by the Cauchy function. Therefore, while determining the physical broadening, the hardware broadening is subtracted from the integral width of the experimental peaks. Considering that, the forms of mathematical functions that describe different types of physical broadening are unknown, thereby different methods were proposed to determine microstructural parameters (crystallite size and microstrain). The analysis of the crystallite size has been carried out using the broadening of XRD peaks. It is known that peak broadening results from both finite crystallite size and strain effect within the crystal lattice [29]. The crystallite size (*D*) has been calculated using the Scherrer method (SM) [30], Williamson-Hall method (WHM), and size-strain plot method (SSPM) [29–33].

**Fig. 2:**
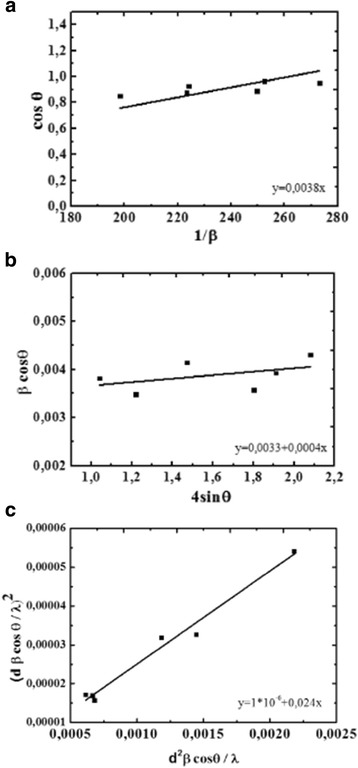
Scherrer plot (**a**), W-H analysis (**b**), and SSP plot (**c**) of Co_1−x_Zn_x_Fe_2_O_4_ ferrites

The particle size in the Scherrer method was determined by the following equation:

1 $$ D=\frac{0.9\lambda}{\beta \cos \theta}\Rightarrow \cos \theta =\frac{0.9\lambda}{D}\left(\frac{1}{\beta}\right) $$

where *D* is the crystallite size (nm), *λ* is the wavelength of X-ray radiation source (1.5406 Å for CuK_α1_), *β* is the integral width, and *θ* is the peak position. Plots were drawn with the (1/*β*) on the *x*-axis and cos*θ* on the *y*-axis (Fig. 2a), and crystallite size *D* was extracted from the slope of fit line. The estimated values of *D* are reported in Table 1. It can be seen that experimental data are not in good agreement with approximation line.

In the Williamson-Hall (W-H plot) method, the XRD peak broadening can be splitted into two parts according to the following expression; *β* = *β*_size_ + *β*_strain_. Assuming that the particle size and strain contributions to line broadening are independent from each other and both have a Cauchy-like profile, the observed line width is simply the sum of the two contributions leading to the Williamson-Hall equation:

2 $$ {\beta}_{\mathrm{hkl}} \cos \theta =\frac{0.9\lambda}{D}+4\varepsilon \sin \theta $$

where *ε* is the strain associated with the nanoparticles. Equation (2) represents a straight line between 4sin*θ* (*x*-axis) and *β*cos*θ* (*y*-axis). The slope of the line gives the strain (*ε*) and the intercept (0.9*λ*/*D*) with *y*-axis gives the crystallite size (*D*) (Fig. 2b). As can be seen from Fig. 2b, the points are widely spread around the fitted line. This obviously indicates that either some other parameters of the studied powders were not taken into account in the used model or that other methods should be used.

There is another model that can be used also to determine the crystallite size (*D*)—the size-strain plot method, which has the advantage that less weight is given to data from reflections at height angles, where precision is usually lower. In this approximation, it is assumed that the “crystallite size” profile is described by the Lorentzian function and the “strain profile” by the Gaussian function:

3 $$ {\left({d}_{\mathrm{hkl}}{\beta}_{\mathrm{hkl}} \cos \theta /\lambda \right)}^2=\frac{K}{D}\left({d}_{\mathrm{hkl}}^2{\beta}_{\mathrm{hkl}} \cos \theta /\lambda \right)+{\left(2\varepsilon \right)}^2 $$

where *K* is constant that depends on the shape of the particles (for spherical particles, *K* = 3/4). In Fig. 2c, where (*d*_hkl_ *β*_hkl_cos*θ*/*λ*)^2^ and (*d*_hkl_)^2^ *β*_hkl_cos*θ*/*λ* were plotted on *x*- and *y*-axes, respectively. In this case, the particle size is calculated from the slope of the linearly fitted data and the root of the *y*-intercept gives the strain. As can be seen from Fig. 2c, all the experimental points are good enough approximation to a straight line.

The average crystallite sizes obtained from the above methods (Table 1) remain in the nanometer regime by means of Zn doping and are within a close range, even though the values obtained by SSPM are slightly lower than those calculated using SM and WHM methods.

Interestingly, the substitution of Co by Zn results in an increase of crystallite size, almost by half for 50% Zn content. This means that Zn favors grain growth during the preparation process of spinel phase. The as-obtained values are in good agreement with the particle sizes estimated from TEM images as can be seen in Table 1.

The ionic packing coefficients *P*_a_ and *P*_b_ at the tetrahedral and octahedral sites, respectively, can be estimated using the following equations [34]:

$$ \begin{array}{cc}\hfill \begin{array}{l}{r}_{\mathrm{xt}}= a\sqrt{3}\left( u-0.25\right)-{R}_{\mathrm{O}}\\ {}\kern2.76em {P}_{\mathrm{a}}=\frac{r_{\mathrm{xt}}}{R_{\mathrm{A}}}\end{array}\hfill & \hfill \begin{array}{l}{r}_{\mathrm{xo}}= a\left(0.625- u\right)-{R}_{\mathrm{O}}\\ {}\kern1.92em {P}_{\mathrm{b}}=\frac{r_{\mathrm{xo}}}{R_{\mathrm{B}}}\end{array}\hfill \end{array} $$

where *r*_xt_ and *r*_xo_ are the interstitial radii whereas *R*_A_ and *R*_B_ are the average values of the ionic radii at the tetrahedral and octahedral sites, respectively, *u* is the anion parameter, *a* is the lattice parameter, and *R*_O_ is the oxygen radius (1.38 Å) [28]. It is claimed [34] that the small values of the ionic packing coefficient, *P*_a_ and *P*_b_, (smaller than 1), testify to the smaller ion distances and larger overlapping of the cation and anion orbitals, suggesting the existence of cation or anion vacancies.

The degree of the ionic packing of the spinel structure can be determined using the fulfillment coefficient of the unit cell, *α*, which can be estimated using the following relation [35]:

$$ \alpha =\frac{32\pi}{3{a}_{exp}^3}\left({r}_{\mathrm{A}}^3+2{r}_{\mathrm{B}}^3+4{R}_{\mathrm{O}}^3\right) $$

The vacancy parameter, *β*, is defined as a normalized volume of the missing ions at the nodal points of the spinel structure [34]. It is a measure of the total vacancy concentration existing in the material and can be estimated using the following equation [34, 35]:

$$ \beta =\left(\frac{a_{\mathrm{th}}^3-{a}_{exp}^3}{a_{\mathrm{th}}^3}\right)\times 100\% $$

**Fig. 3:**
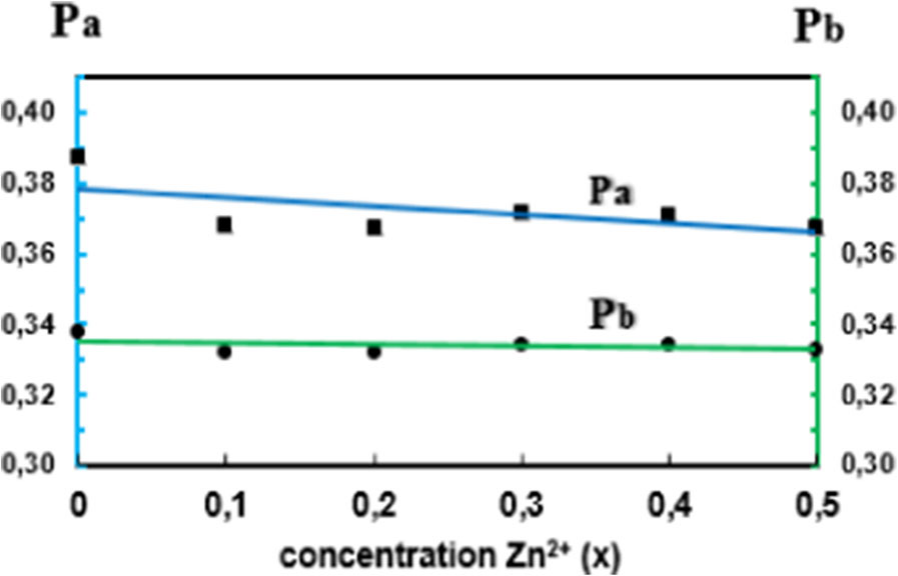
The ionic packing coefficient P_a_ and P_b_ versus Zn(*x*) for the Co_1−x_Zn_x_Fe_2_O_4_ ferrites

**Fig. 4:**
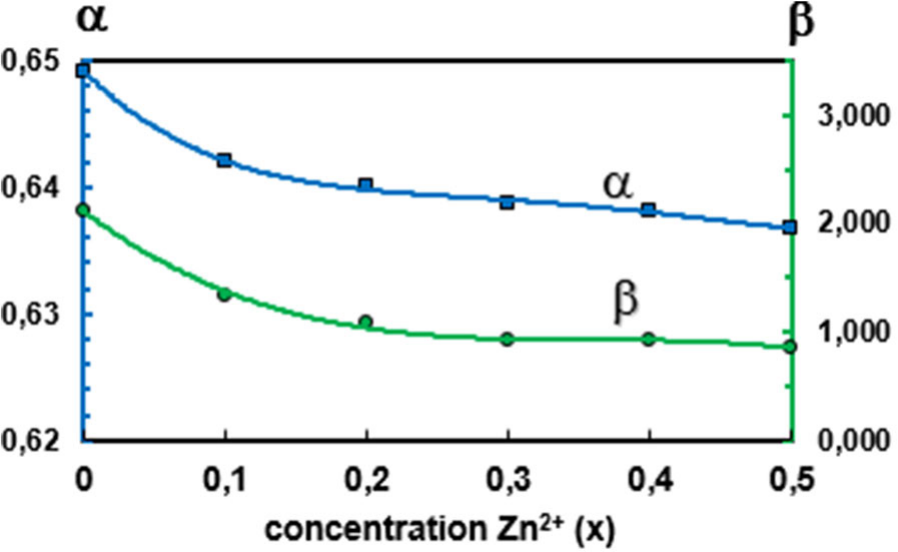
The fulfillment coefficient *α* and vacancy parameter *β* versus Zn(x) for the Co_1−x_Zn_x_Fe_2_O_4_ ferrites

The calculated values *α* and *β* are shown in Table 2 as well as Figs. 3 and 4 as a function of Zn^2+^ concentration. The small values of the fulfillment coefficient *α* (*α* < 1) and the ionic packing coefficient (*P*_a_, *P*_b_ < 1) indicate the presence of vacancies at both tetrahedral and octahedral sites. It can be seen that *P*_b_ decreases while *P*_a_ remains almost constant with increasing Zn content. Also, the fulfillment coefficient *α* decreases to a small extent, while the vacancy parameter *β* decreases more significantly than *α*. The increase in *β* values indicates the presence of cation or anion vacancies.

**Table 2:** Ionic packing coefficient *P*_a_, *P*_b_, fulfillment coefficient *α*, and vacancy parameter *β* for the Co_1−x_Zn_x_Fe_2_O_4_ ferrites

*x* (Zn^2+^)	*r*_xt_ (Å)	*r*_xo_ (Å)	*P*_a_	*P*_b_	*α*	*β* (%)
0.00	0.4367	0.7037	0.387	0.338	0.649	2.119
0.10	0.4843	0.6859	0.368	0.332	0.642	1.329
0.20	0.4890	0.6870	0.368	0.332	0.640	1.090
0.30	0.4848	0.6927	0.371	0.334	0.639	0.913
0.40	0.4869	0.6928	0.371	0.334	0.638	0.916
0.50	0.4960	0.6894	0.367	0.333	0.637	0.853

### Surface Morphology of Co-Zn Ferrites

**Fig. 5:**
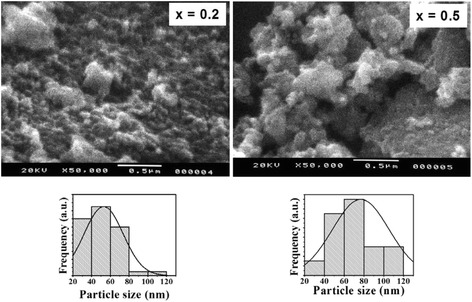
The SEM images and particle size distribution (obtained from TEM) for the samples with *x* = 0.2 and *x* = 0.5 for the Co_1−x_Zn_x_Fe_2_O_4_ spinels

The scanning electron micrographs (SEM) of Zn-doped CoFe_2_O_4_ samples (with *x* = 0.2 and *x* = 0.5) shown in Fig. 5 indicate the formation of agglomerates of very fine particles with almost spherical shape. It can be seen that the Fig. 5 shows heavily concentrated particles of nanoscale regime for Co_0.8_Zn_0.2_Fe_2_O_4_ and Co_0.5_Zn_0.5_Fe_2_O_4_ samples. This is due to its permanent magnetic moment; hence, each particle is permanently magnetized and tends to agglomerate with other particles. The Zn-substituted nanoparticles possess higher magnetic moment leading to more clustering. The average particle size and size distribution obtained from TEM analysis depend on the composition of the ferrite and have a tendency to increase (for example, for *x* = 0.2, average size is equivalent 53 nm while for *x* = 0.5 average size is equivalent 77 nm, Fig. 5). It is evident from TEM images that the powders show almost spherical shape. TEM images confirms that the average particle size is in the range of 46–77 nm (Table 1), which is very close to the values obtained by XRD analysis.

**Fig. 6:**
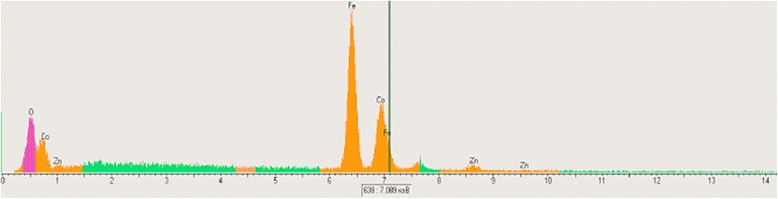
EDS spectra of Zn_0.1_Co_0.9_Fe_2_O_4_

The elemental composition of Co_1−x_Zn_x_Fe_2_O_4_ spinels (*x* = 0, 0.1, 0.2, 0.3, 0.4, 0.5) is obtained from energy dispersive X-ray (EDS) analysis (Table 3), only most representative ones are shown in Fig. 6. The peaks corresponding to Zn, Co, Fe, and O elements are observed in all Zn-doped CoFe_2_O_4_ samples. The sample compositions are taken to be equal to the nominal ones.

**Table 3:** EDS data for the Co_1−x_Zn_x_Fe_2_O_4_ spinels

Zn^2+^ content *x*	Elements (at. %)									
	Theoretical (expected)					Experimental (actual)				
	Co	Zn	Fe	O	Total	Co	Zn	Fe	O	Total
0.00	14.29	–	28.57	57.14	100	14.15	–	28.68	57.17	100
0.10	12.86	1.43	28.57	57.14	100	12.71	2.01	28.22	57.06	100
0.20	11.43	2.86	28.57	57.14	100	11.54	3.13	28.26	57.07	100
0.30	10.00	4.29	28.57	57.14	100	10.90	4.48	27.70	56.92	100
0.40	8.57	5.71	28.57	57.14	100	8.80	5.79	28.33	57.08	100
0.50	7.14	7.14	28.57	57.14	100	6.92	7.87	28.17	57.04	100

### FTIR Spectroscopy and Elastic Properties

**Fig. 7:**
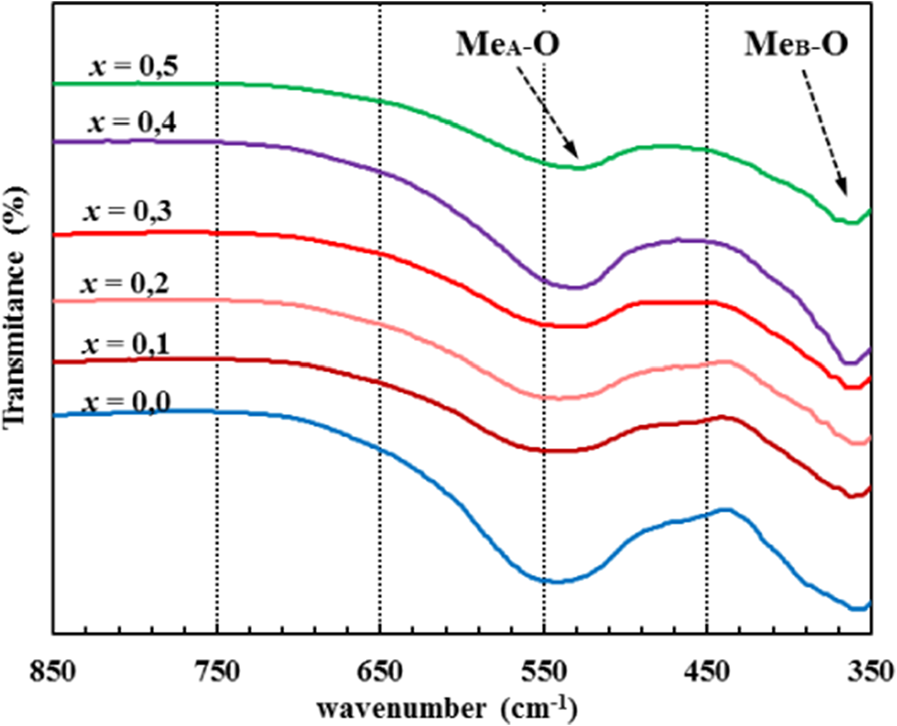
FTIR spectra of Co_1−x_Zn_x_Fe_2_O_4_ ferrites

The FTIR spectra of Co-Zn ferrite samples are shown in Fig. 7. All spectra consist of two main peaks located at about 542–529 cm^−1^ and 360–365 cm^−1^, which confirm the formation of spinel ferrite structure [28]. The ~535 cm^−1^ peak is due to vibration mode of tetrahedral sublattice, while ~363 cm^−1^ peak is due to vibration mode of octahedral sublattice in the spinel structure.

Structural and FTIR data of spinel ferrite are used for the estimation of elastic moduli and the Debye temperature. The Debye temperature of all samples is calculated using the wavenumber of IR bands [36]:


where , *k* is the Boltzmann constant, *C* is velocity of light (*c* = 3 × 10^8^ cm/s), and *υ*_*av*_ is the average wavenumber of bands. The values of the Debye temperature for Co_1-x_Zn_x_Fe_2_O_4_ samples are shown in Table 4. It is observed that the Debye temperature decreases with increasing Zn^2+^ content and can be associated to the decrease in wavenumber of the peak usually attributed to Me-O bond vibration in the tetrahedral site.

**Table 4:** FTIR parameters, the Debye temperature *θ*_*D*_, elastic moduli for the Co_1−x_Zn_x_Fe_2_O_4_ spinels

Zn^2+^ content (*x*)	*υ*_*av*_ (cm^−1^)	*θ*_*D*_ (K)	*E* (GPa)	*G* (GPa)	*B* (GPa)	*σ*
0.00	154.59	649.2	149.11	58.85	106.59	0.27
0.10	155.10	650.3	149.79	59.21	106.15	0.26
0.20	153.35	646.1	153.78	61.77	100.43	0.24
0.30	153.32	646.1	149.71	59.49	103.25	0.26
0.40	153.43	646.1	160.37	65.82	94.86	0.22
0.50	152.58	644.0	165.47	69.57	88.73	0.19

**Fig. 8:**
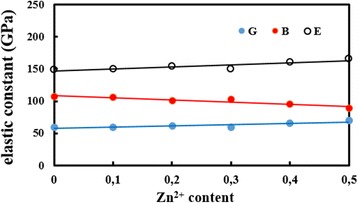
Variation of Young’s modulus (*E*), rigidity modulus (*G*), and bulk modulus (*B*) with Zn content (*x*) in the Co_1−x_Zn_x_Fe_2_O_4_ system

The different elastic moduli for cubic structure are calculated using the standard relations discussed elsewhere [36–38]: Young’s modulus (*E*), rigidity modulus (*G*), bulk modulus (*B*), and Poisson’s ratio (*σ*)*.* The values of these moduli are shown in Fig. 8 and Table 4. Figure 8 shows that with increasing Zn content, all elastic moduli increase except *B*. This behavior of elastic moduli is attributed to the interatomic bonding between various cation within spinel ferrites. The values of Poisson’s ratio for all samples remain almost constant, i.e., in the range 0.19–0.27. It has been reported that a value that lies within the range between −1 and 00.5 implies a good elastic behavior and is in accordance with the theory of isotropic elasticity [36, 38]. This value of the Poisson ratio is in good agreement with Al-substituted Mn_0.5_Zn_0.5_Fe_2_O_4_ ferrite [38].

### Optical Properties (Diffuse Reflectance Spectroscopy)

**Fig. 9:**
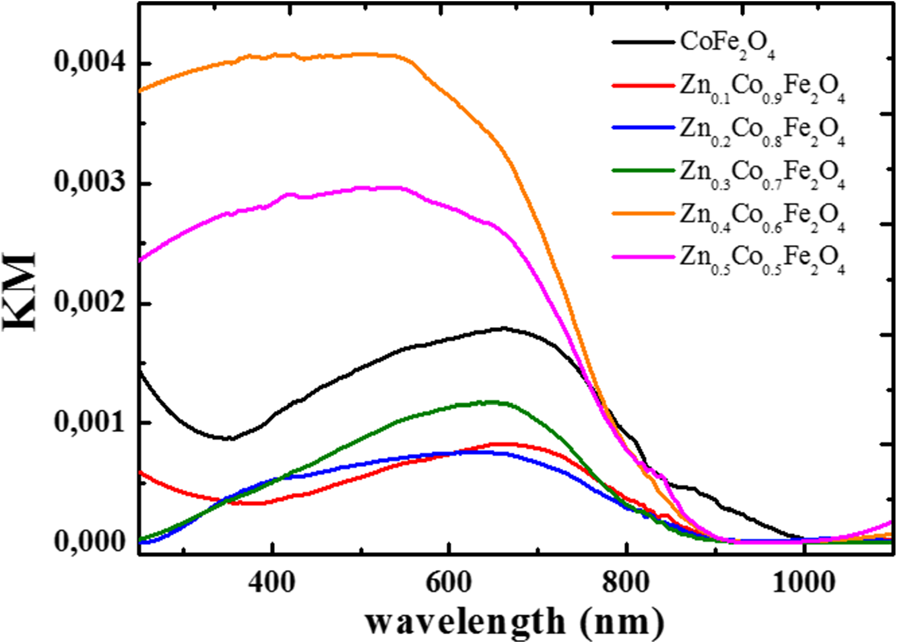
UV–vis spectra presented as the Kubelka-Munk function for Zn_x_Co_1−x_Fe_2_O_4_ system as a function of Zn content

Diffuse reflectance spectra have been recorded and transformed to the Kubelka-Munk function (Fig. 9). The Kubelka-Munk function is the conversion of the sample reflectance, defined as:

$$ \mathrm{K}\mathrm{M}=\frac{{\left(1- R\right)}^2}{2 R} $$

where *R* is absolute reflectance. In order to determine the band gap energy of the material, it is necessary to use the Tauc plot. The band gap can be estimated using the following equation:

$$ {\left(\alpha v\right)}^{\frac{1}{n}} \propto h v\hbox{--} {E}_{\mathrm{BG}}, $$

where *α*, *h*, *v*, *E*_BG_, and *n* are absorption coefficient, the Planck constant, oscillation frequency, band gap energy, and constant relating to a mode of transition, respectively. The constant *n* is $\frac{1}{2}$ for allowed direct transition and 2 for indirect transition.

**Fig. 10:**
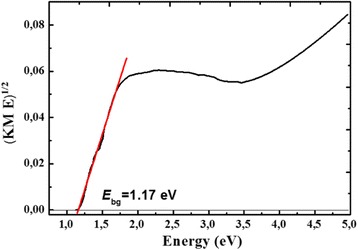
Tauc plot for indirect band gap CoFe_2_O_4_

**Fig. 11:**
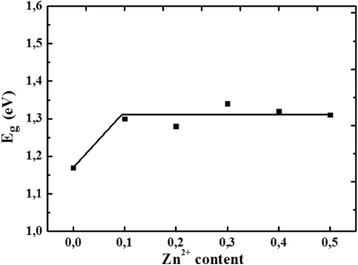
Band gap energy of Zn_x_Co_1−x_Fe_2_O_4_ system as a function of Zn content (error ±0.03 eV)

All materials absorb up to ca. 900 nm, except CoFe_2_O_4_ which absorbs up to 1000 nm. For higher Zn content (*x* = 0.4–0.5), a stronger absorbance within the UV–vis range is observed. The Tauc transformation of spectra enables the determination of optical band gap energies (*E*_bg_). An example of the Tauc plot, derived from UV–vis DRS spectrum, is presented in Fig. 10 for CoFe_2_O_4_ (indirect semiconductor). By checking the linearity of the plot of ${(av)}^{\frac{1}{n}}$ vs. *hv*, it is possible to determine the band gap energy as the *x*-intercept of the extrapolated linear fits. This procedure is commonly used for semiconductors characterization and is well described in literature [39–42]. It can be noticed that the substitution of Co by Zn results in an increase of *E*_bg_ from 1.17 eV for CoFe_2_O_4_ to 1.30 ± 0.03 eV for Zn_0.1_Co_0.9_Fe_2_O_4_, then it becomes almost constant (Fig. 11).

### VSM Measurements

**Fig. 12:**
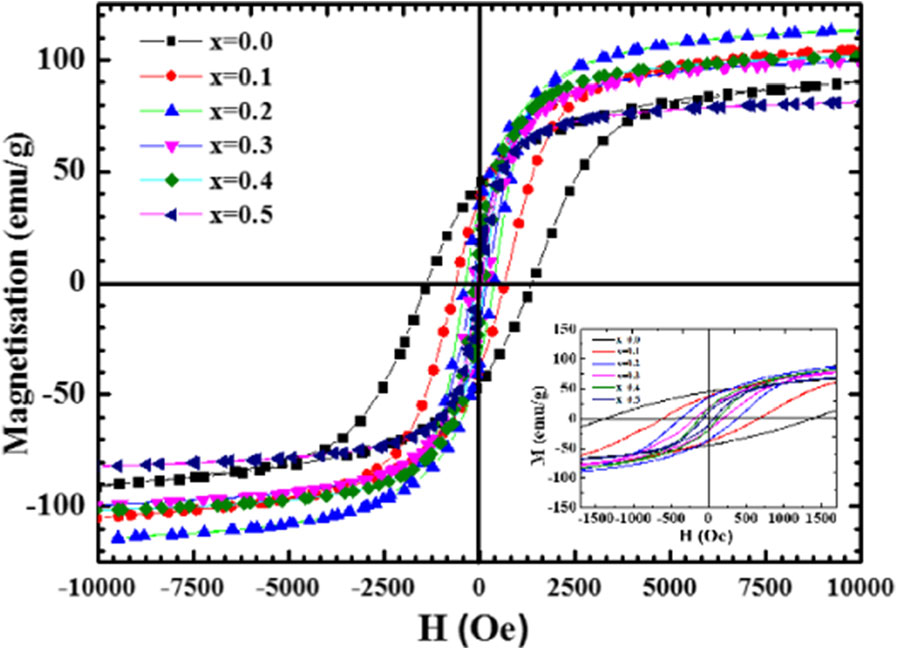
Saturation magnetization (M_s_) versus applied magnetic field (H) of the Co_1−x_Zn_x_Fe_2_O_4_ samples at room temperature (*Inset* low field region of the loops)

**Fig. 13:**
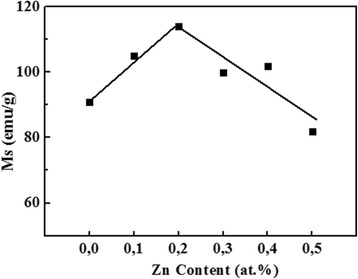
Variation of the saturation magnetization (M_s_) of the Co_1−x_Zn_x_Fe_2_O_4_ (*x* = 0.0, 0.1, 0.2, 0.3, 0.4, and 0.5) system versus Zn content

CoFe_2_O_4_ shows a ferromagnetic behavior with a large hysteresis loop (Fig. 12). Doping with Zn ions reveals also a ferromagnetic behavior while induces important modifications in the magnetic properties; the hysteresis loop decreases drastically with Zn content (Table 5). M_s_ was found to increase with Zn to reach an optimum value of 114 emu/g for 20% Zn content and then decrease to 82 emu/g for 50% Zn content (Fig. 13). At lower concentrations, Zn ions occupy preferentially tetrahedral A sites of CoFe_2_O_4_ whereas for higher concentration, Zn ions have the tendency to move to octahedral B sites (Fe). It is surprisingly interesting that the substitution of magnetic Co (*μ*_B_ = 3) with a nonmagnetic Zn (*μ*_B_ = 0) results in a 25% increase in M_s_ value.

**Table 5:** Magnetic parameters (saturation magnetization M_s_, remanent magnetization M_r_, coercitivity H_C_) at room temperature of Co_1−x_Zn_x_Fe_2_O_4_ system as function of Zn content (*x*)

*x* (Zn^2+^)	*D* (nm)	M_s_ (emu/g)	M_r_ (emu/g)	H_C_ (Oe)
0	51	91	44	1382
0.1	46	105	38	628
0.2	53	114	36	377
0.3	55	100	19	188
0.4	69	102	18	119
0.5	77	82	10	75

**Fig. 14:**
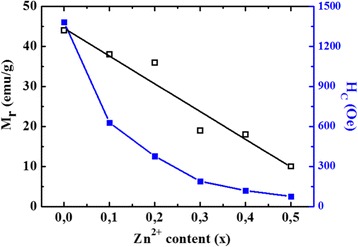
Variation of the remanent magnetization (M_r_) and coercivity (H_C_) of the Co_1−x_Zn_x_Fe_2_O_4_ (*x* = 0.0, 0.1, 0.2, 0.3, 0.4, and 0.5) system versus Zn content

H_C_ is found to decrease with increasing the concentration of Zn. H_C_ decreases drastically by more than 50% with only 10% of Zn, while M_r_ decreases linearly and gradually with Zn content reaching a reduction of 77% for 50% Zn substitution in comparison with pure CoFe_2_O_4_ (Fig. 14).

**Fig. 15:**
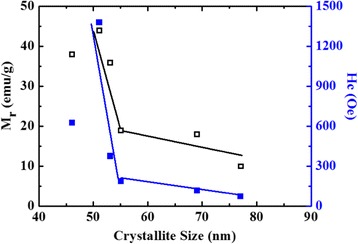
Variation of the remanent magnetization (M_r_) and coercivity (H_C_) of the Co_1−x_Zn_x_Fe_2_O_4_ (*x* = 0.0, 0.1, 0.2, 0.3, 0.4, and 0.5) system versus crystallite size (from TEM)

It is reported that at particular range of grain size, H_C_ and M_r_ become highly sensitive to the change in grain size [43], which is consistent with the presented results. At smaller ranges of crystallite size (*D*), H_C_ and M_r_ showed a rapid decrease as *D* increases, while a gradual decrease is noticed as *D* gets larger. This relation is significant for the Zn-doped CoFe_2_O_4_ (Fig. 15).

## Conclusions

Zn-doped CoFe_2_O_4_ NPs have been successfully synthesized via chemical co-precipitation route. XRD and FTIR confirmed the formation of single cubic spinel phase. Doping with Zn showed a considerable effect on structural, spectral, and magnetic properties. The crystallite size and lattice parameter increase gradually while increasing Zn content. This can be associated with ionic radii (Zn is larger than Co) and that Zn favors grain growth. The line broadening was analyzed by the Scherrer formula, W-H analysis, and the SSP method. The TEM results were in good agreement with the results of the SSP method. SEM analysis showed spherical-shaped particles forming agglomerates. The energy gap (*E*g) is found to increase for 10% Zn and then remains constant for higher doping level. Magnetic measurements reveal a ferromagnetic behavior while the hysteresis loop tends to decrease with Zn concentration. M_s_ is found to be sensitive to Zn concentration, while M_s_ and H_C_ decrease dramatically with increasing the amount of Zn.

## Abbreviations

DRS: Diffuse reflectance spectroscopy
EDS: Energy dispersive spectroscopy
FTIR: Fourier transform infrared spectroscopy
H_C_: Coercivity
M_r_: Remanent magnetization
M_s_: Saturation magnetization
NPs: Nanoparticles
SEM: Scanning electron microscopy
SF: Spinel ferrite
SM: Scherrer method
SSPM: Size-strain plot method
TEM: Transmission electron microscopy
VSM: Vibrating sample magnetometer
WHM: Williamson-Hall method
XRD: X-ray diffraction
